# Concentrated Medicines

**Published:** 1863-04

**Authors:** 


### CONCENTRATED MEDICINES.

By reference to our advertising sheet it will be observed that O. H. P. Champlin is Agent in this City for the well known firm of Jas. R. Nichols & Co., of Boston, Manufacturing Chemists and Wholesale Druggists, who manufacture Nitrate Silver, Chloride Gold, Elixir Bark and Iron, Protoxide of Iron, Citrate of Iron, Iodide of Lime, Iodoform, Propylamin, Proteine, Hypophosphite Salts and Syrups, Ethers, Chloroform, Acids, and all the fine Chemicals used by druggists, artists, manufacturers and experimenters.

We have recently been prescribing some of these preparations, which are exceedingly well made and pure to all appearance. The Iodide of Lime we are using in some cases in place of Iodide of Potassium, and find it much more palatable, and less expensive to the patient. We cannot yet speak of its therapeutic effect, but will be able to speak more positively upon the subject after a little more extended trial. We shall take occasion hereafter to give the results of our experience in the practical use of many of these very beautifully prepared medicines.

